# FLIMExplorer: interactive GUI for object-based visualization and analysis of fluorescence lifetime images

**DOI:** 10.1088/2515-7647/ae7da3

**Published:** 2026-07-07

**Authors:** Blanche ter Hofstede, Samantha Morganti, Daniela De Hoyos Canales, Anna Theodossiou, Amanda Galloway, Alex J Walsh

**Affiliations:** 1 Biomedical Engineering, Texas A&M University, College Station, TX, United States of America; 2 Bioengineering, Rice University, Houston, TX, United States of America

**Keywords:** image processing, statistical analysis, single cell image analysis, cellular heterogeneity, multiphoton microscopy, fluorescence lifetime imaging microscopy

## Abstract

Fluorescence lifetime imaging microscopy (FLIM) enables non-invasive measurement of cellular metabolism with single-cell and subcellular resolution, providing advantages over traditional population-level metabolic assays. However, current single-cell FLIM analysis workflows typically rely on multiple software tools for lifetime extraction, segmentation, and downstream analysis, often resulting in convoluted workflows and a disconnect between quantitative measurements and image context, limiting transparent quality control. Here, we present FLIMExplorer, an interactive, Python-based tool for downstream single-cell FLIM analysis and visualization. FLIMExplorer links quantitative FLIM endpoints and their underlying image objects, enabling users to explore key FLIM features (NAD(P)H and FAD: *τ*_m_, *τ*_1_, *τ*_2_, and *α*_1_) at the single-cell level, visualize image objects corresponding to individual data points, and perform statistical comparisons across experimental groups within a single graphic user interface app. FLIMExplorer handles inputs of both (1) pixel-level FLIM outputs generated by upstream fitting software and (2) pre-processed, cell-average FLIM datasets. By integrating visualization, quality control, and statistical analysis at the single-cell level within a single platform, FLIMExplorer improves the integration of quantitative results with image context in downstream FLIM analysis.

## 1 Introduction

Fluorescence lifetime imaging microscopy (FLIM) is a powerful imaging technique that measures the fluorescence lifetime of endogenous and exogenous fluorophores [1–4]. FLIM enables non-invasive measurement of cellular metabolism and coenzyme binding using the autofluorescence of nicotinamide adenine (phosphate) dinucleotide (NAD(P)H) and flavin adenine dinucleotide (FAD) [5–7]. Unlike population-level metabolic assays, such as the Seahorse assay [8], FLIM allows analysis at the single-cell and subcellular levels.

Time-domain autofluorescence FLIM data are typically analyzed using a biexponential decay model to extract the lifetimes and fractional contributions of free and protein-bound NAD(P)H and FAD coenzymes [6, 7, 9]. These FLIM-derived endpoints are continuous variables that report metabolic heterogeneity between and within individual cells [10]. When performed on multiphoton (MP) or confocal systems with high spatial resolution, FLIM images can be segmented into individual cells or subcellular features which enables quantitative, object-based metabolic analysis [11–13]. Accordingly, specialized software tools are needed to support object-based quantification and statistical analysis of FLIM datasets.

Current single-cell FLIM analysis workflows often rely on multiple software tools for lifetime extraction, image segmentation, and downstream analysis. Upstream FLIM processing can be performed using commercial software such as SPCImage NG (Becker & Hickl) [14] and SymPhoTime 64 (PicoQuant) [15], as well as open-source software such as FLUTE [16], FLIMfit [17], FLIMview [18], the ImageJ plugin FLIMJ [19], and Napari-Live-FLIM [20]. Of note, FLIM Playground integrates FLIM fitting and analysis into a single, interactive, open-source software kit [21], a step toward streamlining and standardizing the FLIM workflow.

Despite growing interest in single-cell analysis to study cellular heterogeneity in disease states and drug response, and advances in automated image segmentation software such as Cellpose [22], software tools for downstream cell-level FLIM analysis remain limited. Existing FLIM analysis software typically output image-level metrics or phasor coordinates, leaving a disconnect between quantitative data and the corresponding image objects. As a result, users must manually perform cell-level data extraction, outlier assessment, and comparative analysis across heterogeneous populations. While this can be accomplished using general image-analysis platforms such as ImageJ [23] and CellProfiler Analyst [24], these workflows require extensive manual handling of per-object results, resulting in a cumbersome, user-intensive process. To address similar challenges, some groups have developed custom scripts or field-specific tools such as QuPath, which is optimized to map segmented objects with the quantitative data and corresponding images for pathology image analysis [25].

We present FLIMExplorer, an interactive, Python-based tool for single-cell FLIM analysis and visualization. FLIMExplorer maintains links between quantitative FLIM measurements and their corresponding image. FLIMExplorer enables users to explore key FLIM features (NAD(P)H and FAD *τ*_m_, *τ*_1_, *τ*_2_, and *α*_1_) at the single-cell level, visualize image objects corresponding to individual data points, and perform statistical comparisons across experimental groups within a unified interface. The software was tested on SPCImage ASCII outputs from two different MP FLIM microscopes using three different datasets: T cell data, macrophages isolated from whole blood, and a dose-response, time course study of breast cancer cell data. By supporting interactive inspection and biologically justified handling of outliers, FLIMExplorer contributes to improved integration of quantitative results and image context in downstream FLIM analysis. A summary of existing software relevant to FLIM analysis is shown in table 1. Due to its modularity, this software can be adapted in the future to be generalizable for other input FLIM files and include additional FLIM processing steps.

**Table 1.** Comparison of commercial and open-source software for time-domain FLIM analysis including upstream and downstream analysis techniques. Statistical analysis refers to downstream inferential statistical workflows. ✓ indicates the tool has capabilities and ✓✓ indicates main function of software.

				Upstream analysis		Downstream analysis				
Software	FLIM-data handling	FLIM acqusition	ROI/single-cell analysis	FLIM fitting	Phasor analysis	Statistical analysis	Global data visualization	Image-data linkage	QC/outlier management	Open source
SPCImage NG [14]	**✓**	**✓**	**✓** Manual or threshold-based ROI selection	**✓**	**✓**	—	—	—	—	—
SymPhoTime 64 (PicoQuant) [15]	**✓**	**✓**	**✓** Manual ROI-based selection	**✓**	— Supports external	—	—	—	—	—
FLUTE [16]	**✓**	—	—	—	✓✓	—	—	—	—	**✓**
FLIMfit [17]	**✓**	—	**✓**	**✓✓**	**—**	—	**✓**	—	—	**✓**
FLIMJ [19]	**✓**	—	**✓** Can be combined with ImageJ-based ROI manager	**✓✓**	—	—	—	—	—	**✓**
Napari-live-FLIM [20]	**✓**	**✓**	**✓** Can be combined with napari-based segmentation plugins	**✓**	**✓**	—	—	—	—	**✓**
FLIM Playground [21]	**✓**	—	**✓**	**✓**	**✓**	**✓**	**✓**	—	—	**✓**
CellProfiler Analyst [24]	—	**—**	**✓**	—	—	**✓**	**✓**	**✓**	**✓**	**✓**
FLIMExplorer	**✓**	**—**	**✓**	—	—	**✓**	**✓**	**✓**	**✓**	**✓**

## 2 Software description

### 2.1 Overview

**Figure 1.**
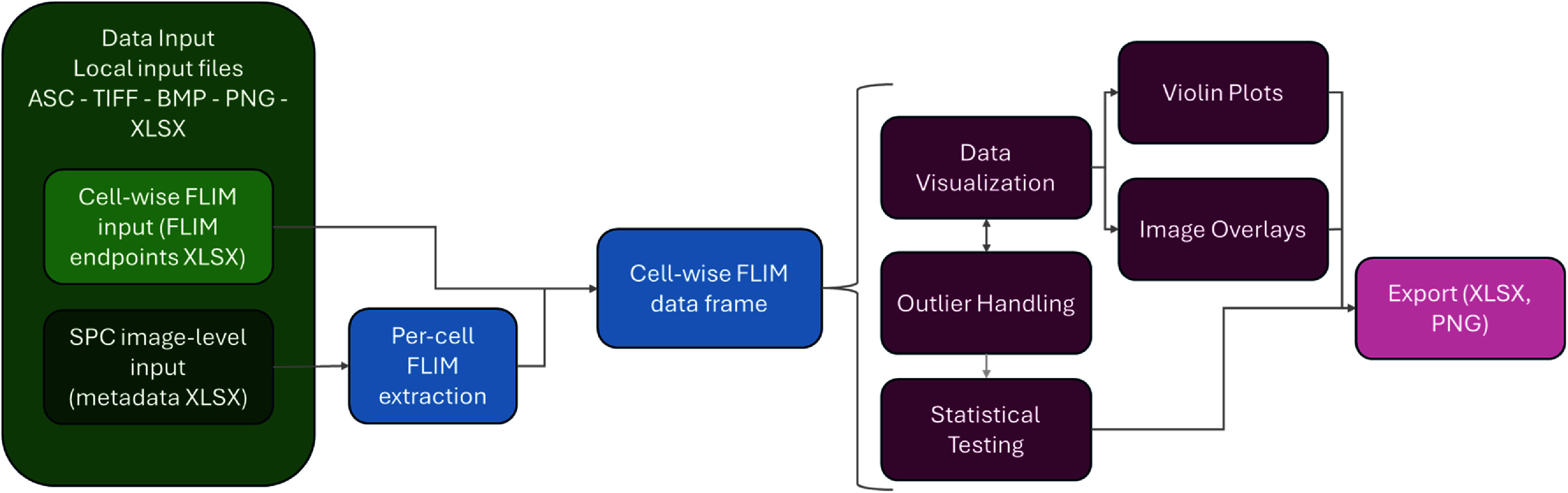
FLIMExplorer workflow diagram.

FLIMExplorer is an open-source, interactive graphical user interface (GUI) for downstream single-cell analysis, visualization, and linkage of FLIM data (figure 1). This software supports data visualization, statistical analysis, and outlier identification and removal within a reproducible workflow. It is implemented in Python using the Dash web framework, allowing the user to operate locally in a web-based GUI.

To facilitate data inspection and quality control, FLIMExplorer provides tools to interactively select individual data points within plots, visualize the corresponding segmented objects in the original FLIM images, and flag or exclude objects as needed. Since automated or semi-automated segmentation methods often include non-cellular objects or dying cells that are difficult to identify from population-level summaries, this interactive linkage between quantitative metrics and image context supports biologically justified outlier handling and transparent quality control of single-cell datasets.

The main features of FLIMExplorer are (1) linking of quantitative FLIM metrics and original image data sets using object masks, (2) performing statistical tests across different experimental groups, and (3) user-guided identification of outliers, all within a user-friendly GUI for experimental researchers.

### 2.2 Architecture and implementation

**Figure 2.**
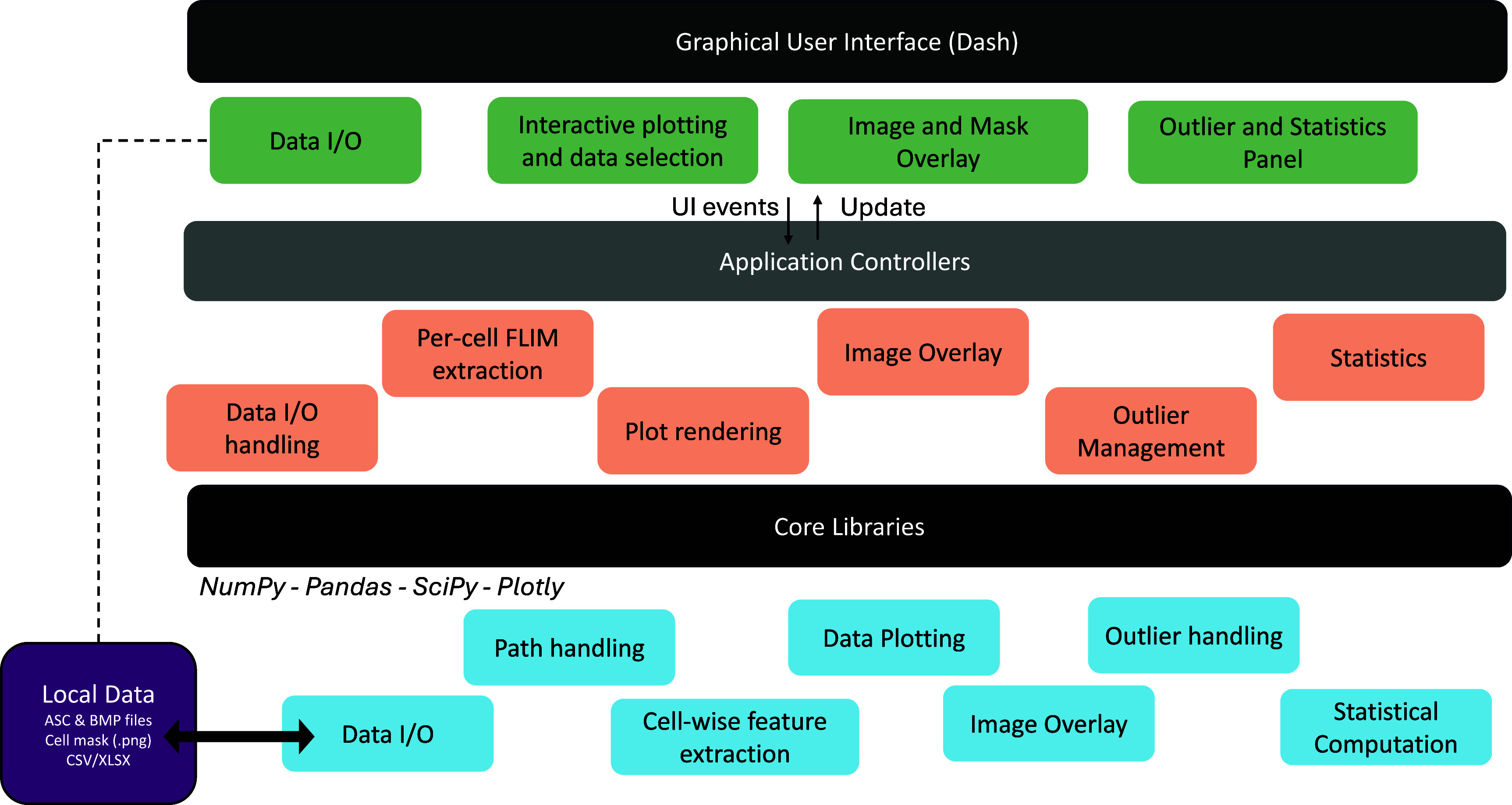
FLIMExplorer layered architecture. The GUI front-end allows the user to interact with controls and plots, and the application controllers manage UI events. Core libraries take local data and result in an updated interface.

FLIMExplorer was developed using a modular, layered architecture (figure 2) in which data import and handling, user interaction, visualization, image rendering, statistical analysis, and data export are implemented as separate modules. This allows for easier maintenance, reusability, and debugging. The GUI front-end allows users to upload data, interact with plots, and customize outlier and statistical analysis parameters. User interface events are handled through Dash application controllers that interface with core analysis libraries to update the UI dynamically. FLIMExplorer is implemented in Python (>3.10) with the Dash framework to support interactive web-based visualization, with Plotly used for figure rendering. Numerical and statistical operations are performed using NumPy, SciPy, pandas, and statsmodels libraries. Image input and processing are handled using imageio in combination with NumPy arrays.

Core functionality is organized into the following seven modules:

1 Data I/O: Handles import of tabular data from CSV or Excel files with automatic type inference and initialization of required metadata (outlier flags). Supports loading of ASCII FLIM images and PNG integer cell masks, extraction of pixel-level FLIM endpoints into per-cell values for NAD(P)H and FAD, and export of updated cell-level data of single-cell FLIM endpoint results of image-level metadata.
2 Cell-wise feature extraction: Calculates optical redox ratio (${\mathrm{ORR}} = {I_{{\mathrm{FAD}}}}/\left( {{I_{{\mathrm{NAD}}\left( {\mathrm{P}} \right){\mathrm{H}}}} + {I_{{\mathrm{FAD}}}}} \right)$) and the fluorescence lifetime redox ratio (FLIRR = $\left( {1 - {\alpha _{1,{\text{ NAD}}\left( {\mathrm{P}} \right){\mathrm{H}}}}} \right)/{\alpha _{1,{\text{ FAD}}}}$). Generates a structured pandas DataFrame containing formatted FLIM endpoints, experimental conditions (e.g. treatment groups, time points), cell indices within image, and image identifiers.
3 Path handling: Allows the user to define file name patterns and connect image and mask files with row-level metadata or FLIM-processed data without code modification.
4 Data plotting: Generates violin plots with support for primary grouping variables, and secondary independent variables (hue). Row-level identifiers are embedded within Plotly custom data field to maintain bidirectional linkage between plotted data points and the underlying dataset.
5 Image overlay: Renders NAD(P)H and FAD fluorescence intensity and FLIM color images (user-specified; e.g. *τ*_m_), extracts cell outlines from PNG integer cell masks, and renders overlay images using a headless Matplotlib backend.
6 Outlier handling: Manages user-driven outlier identification by storing and updating outlier flags for individual cells, enabling reversible inclusion or exclusion of data points during visualization and statistical analysis.
7 Statistical computation: Performs pairwise statistical comparisons with adaptive test selection, customizable multiple comparison correction, and optional customizable plot annotations of statistical significance on plots.

### 2.3 Functionality

**Figure 3.**
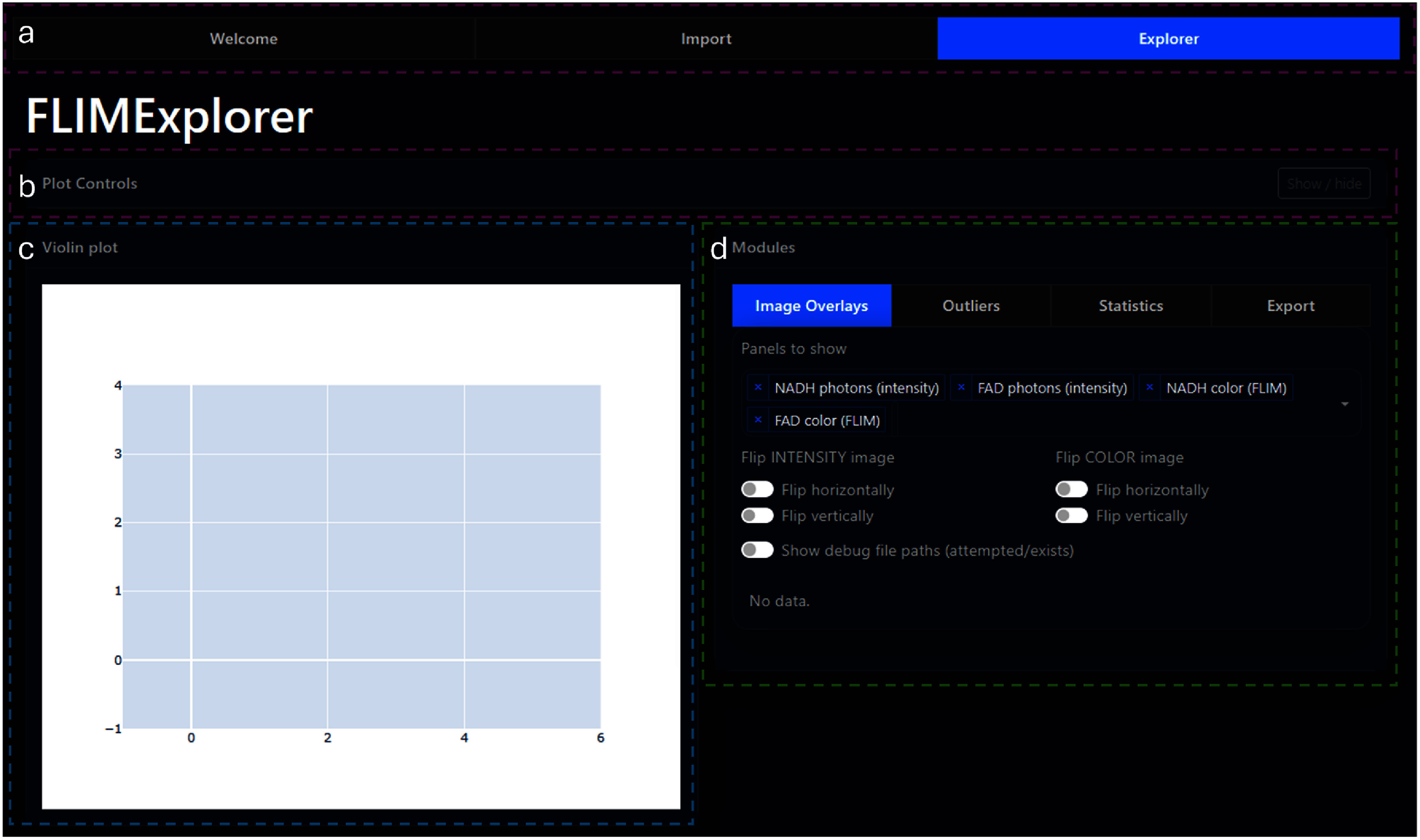
FLIMExplorer GUI layout: explorer page. (a) Tabs for welcome page, data import page, and main explorer pages. (b) Plot controls for selecting metrics and variables to be analyzed, and customization of plot, including font size, font, and color palette. (c) Data visualization that displays customizable violin plots with interactive point selection. (d) Image overlay module for visualizing the selected data points and adjusting overlay display settings.

The Explorer page is the main section of the FLIMExplorer software (figure 3) and is used for visualization and analysis of FLIM data following data upload. The following section describes the main components and functionality of this interface. A detailed user manual is available on the project’s GitHub repository.

#### 2.3.1 Data input/output

**Figure 4.**
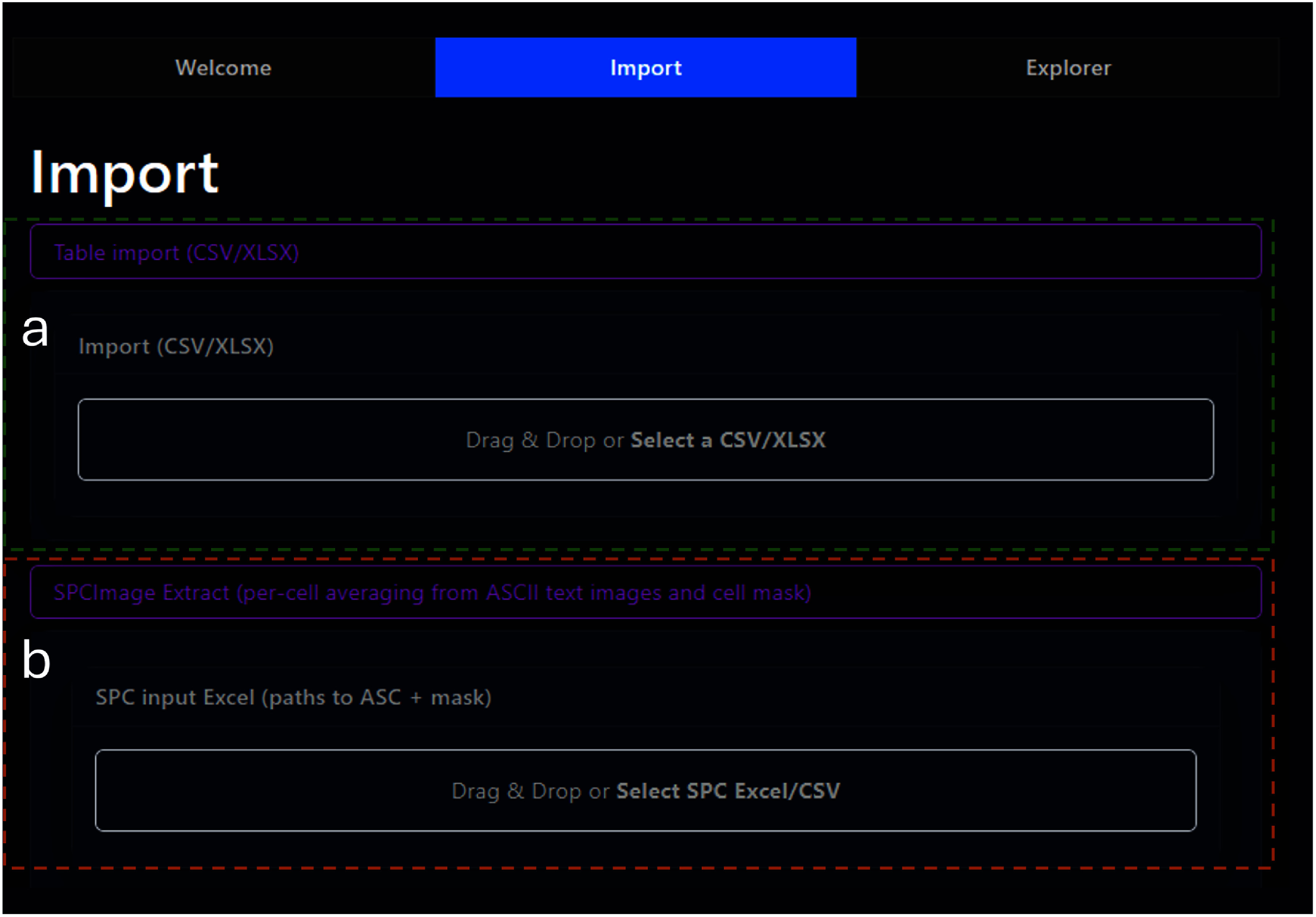
FLIMExplorer GUI layout: importing data. Data input with (a) data upload of pre-processed FLIM endpoint data and (b) SPCImage import for per-object extraction using SPCImage derived ASCII text files and cell masks.

FLIM data can be loaded locally in the import page as either 1) pre-processed object-level FLIM data provided in an Excel document (figure 4(a)) or 2) ASCII text files for per-object FLIM endpoint extraction (figure 4(b)). When using FLIM fitting software-generated ASCII files (e.g. SPCImage), an accompanying XLSX metadata file must be uploaded to specify the file stem names of the NAD(P)H and FAD file pairs, the corresponding mask file names, folder paths, and any independent categorical variables (e.g. treatment group, time point). The user can modify the ASCII text image file name pattern in this module.

For pre-processed datasets, an Excel document containing tabulated cell-level FLIM endpoints, cell mask identifiers per image, and associated NAD(P)H and FAD file names must be uploaded. To support standardized data formatting, a supplemental GUI for automatic Excel file generation, along with an example input XLSX file, is provided in the project’s GitHub repository. After import and processing, cell-level results, including outlier flags, can be exported using the Export module.

#### 2.3.2 Violin plots and data point interaction

Once data is loaded, in the main FLIMExplorer page (figure 3(a)), users can select FLIM metrics along with primary and optional secondary variables from a dropdown menu for visualization (figure 3(b)). The primary data visualization consists of violin plots displaying FLIM-derived metrics across experimental groups (figure 3(c)). Hover tooltips are configured to display additional FLIM features (ORR, FLIRR, NAD(P)H, and FAD FLIM endpoints), enabling rapid contextual inspection of individual data points.

#### 2.3.3 Image and mask overlays

Fluorescence intensity and FLIM color images (user-specified, e.g. *τ_m_*) are loaded based on file paths specified by the imported Excel file and are displayed upon selection of corresponding data points (figure 3(d)). The user can specify which image types to show and define which file formats are required for image loading. Mask overlays are rendered on top of intensity and FLIM images to outline the selected cell(s) in red. To ensure proper alignment between the FLIM and intensity images, the plot overlay module provides optional vertical and horizontal transformations for image registration.

#### 2.3.4 Outlier management

**Figure 5.**
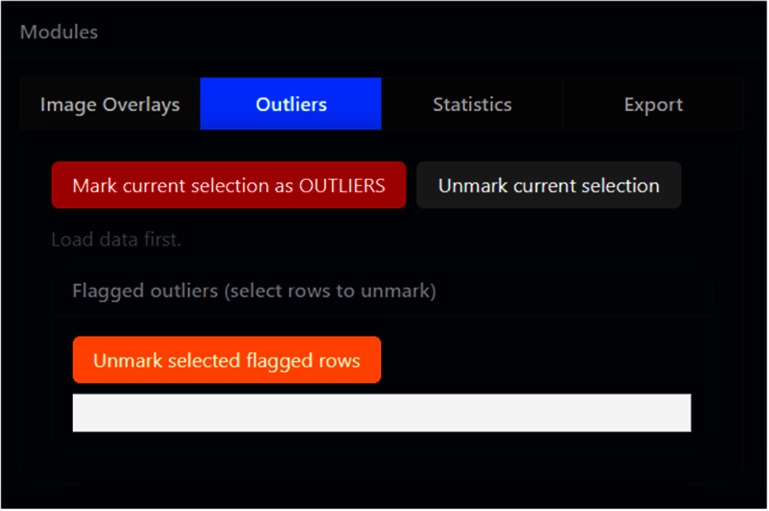
FLIMExplorer GUI layout: outliers. Data points selected on the violin plot can be flagged as outliers. A table is populated with flagged outliers and removed from statistical analysis if desired. Specific outliers can be selected in the table for unflagging.

**Figure 6.**
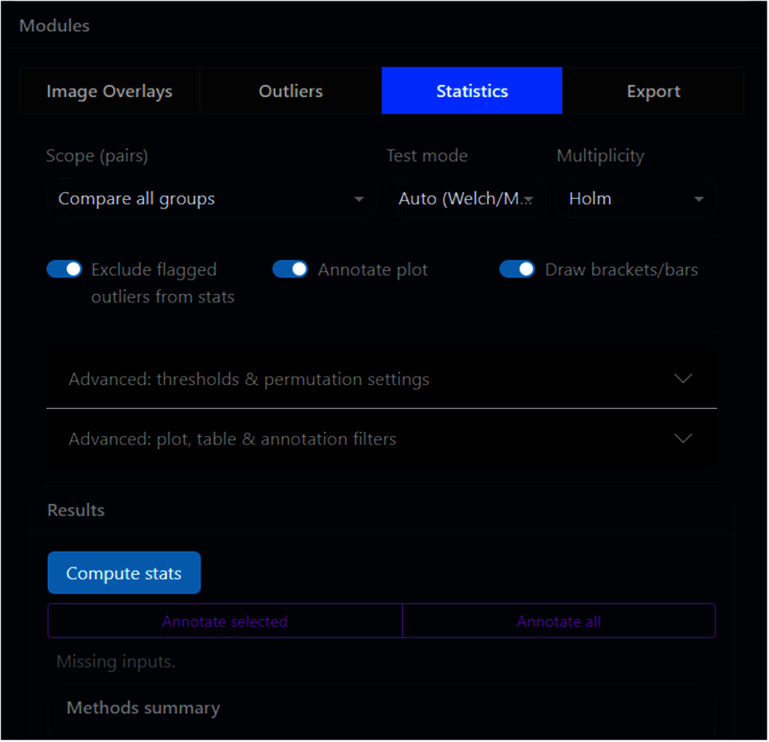
FLIMExplorer GUI layout: statistics. Multiple group comparison statistical testing can be customized and performed, allowing for annotation of significant pairs on the violin plots. The summary and results of the statistical tests are displayed in the Results section.

Selected cells can be marked as outliers or unmarked in the outlier tab (figure 5). Flagged data points are shown in this tab to help the user track all marked outliers and export them via the Export module. An option to exclude these outliers from statistical testing is included in the statistics tab (figure 6).

#### 2.3.5 Statistical analysis

The Statistics Tab (figure 6) enables the user to run statistical testing on the FLIM-derived metrics. Test mode includes Auto, which selects the appropriate test based on normality and sample size, as well as the Welch’s *t*-test, the Mann–Whitney *U* test, and permutation testing for larger data sets. Multiple comparison correction methods (Bonferroni, Holm–Bonferroni, Šidák, and Benjamini-Hochberg) can be selected from a dropdown menu. The significance level (*α*) can be modified by the user in the advanced settings. Once the statistical testing is run, a methods summary is generated, along with a table of statistical results and test selected for each comparison. For analysis involving secondary independent variables, the user can select to compare across all groups, across secondary groups (hues), or within secondary groups (hues).

The statistics module also supports configurable annotation of the violin plot with significance bars and asterisks denoting the pairwise comparison *p*-values. Interactive hover tooltips provide a summary of the statistical test applied to each comparison.

#### 2.3.6 Limitations

This software is optimized for FLIM datasets generated from SPCImage as ASCII text image outputs. Testing was done on images acquired by two different MP FLIM systems to demonstrate compatibility across microscope systems. Other inputs of pixel maps for lifetime values and lifetime fractions must be converted to ASCII text images to process. Therefore, the software allows input of pre-processed, averaged FLIM values per cell, and future iterations of the software may allow for more generalizability for outputs of software, such as FLIMfit and commercial fitting software, that have specific file formats.

File formats and naming conventions vary across FLIM acquisition systems, fitting software packages, labs, and individuals within labs. Because users may organize datasets in various ways, FLIMExplorer requires that the file locations and stem names of paired NAD(P)H and FAD images be explicitly provided in the imported data file to maintain unambiguous data linkage. A supplemental GUI has been developed to customize this import data file. Users may still need to reorganize their data prior to importing it to the GUI.

In its current format, FLIMExplorer does not support alternative file formats or non-FLIM imaging applications. However, the modular architecture of the software allows these assumptions to be relaxed or adapted in future iterations, enabling use for other imaging applications with minimal restructuring.

### 2.4 Key features

• Flexible data import supporting extraction of SPCImage-processed FLIM text images as well as analysis of preprocessed, cell-averaged FLIM data.
• Interactive visualization for data exploration, linking quantitative FLIM metrics to their corresponding image objects.
• Customizable pairwise statistical analysis, allowing for multiple comparison correction and plot annotation with statistical results.
• Outlier management, enabling transparent quality control and reproducible handling of single-cell data.

### 2.5 Technical description

The software is distributed as a compressed archive containing the main application script (app.py) and a YAML configuration file specifying the required Python version (3.10) and all dependent libraries needed to create a dedicated virtual environment. When executed from the command line, the application launches as a local server that hosts an interactive browser-based GUI.

Data processing and visualization is performed locally, and all files (ASCII files, FLIM intensity and color images, cell-level files XLSX/CSV) are accessed directly from the user’s file system. To enable proper data loading and linkage, the user is required to provide an input XLSX metadata file along with the NAD(P)H and FAD data and the corresponding mask file directory path and file name.

## 3 Use cases and benchmarking

FLIMExplorer was tested on multiple time-domain datasets acquired using different MP imaging systems to validate software functionality and generalizability for single-cell FLIM analysis workflows. Dataset 1 consists of MCF10A cells treated with varying concentrations of an LDHB inhibitor drug (AXKO-0046, MedChemExpress) at three time points over 24 h [26]. The GUI was originally designed based on the organization structure of this dataset and per-cell FLIM metrics were extracted using the FLIMExplorer Import page. Dataset 2 consisted of macrophage cell samples isolated from whole blood, with residual red blood cells (RBCs) and platelets remaining following isolation. This dataset was acquired on the same microscope as Dataset 1 by a different user. Previously extracted per-cell FLIM endpoints were imported to FLIMExplorer directly as an Excel file. Dataset 3 was previously published data [27] that was acquired by a third user using a different MP imaging system, to demonstrate the generalizability of FLIMExplorer across users, sample types, and imaging platforms. All input ASCII text files were generated from SPCImage NG (Becker & Hickl) biexponential decay fitting of time-domain FLIM data. Together, these datasets demonstrate the compatibility with multiple experimental designs, imaging systems, users, and FLIM analysis workflows.

### 3.1 Dataset 1: MCF10A cells

#### 3.1.1 Cell preparation and imaging parameters

The first dataset used for testing consists of four treatment groups (0.1 *μ*M, 1.0 *μ*M, low-dose vehicle, high-dose vehicle) of MCF10A cells treated with LDHB inhibitor AXKO-0046 and imaged at three time points (1 h, 12 h, and 24 h). Data were collected using a custom-built MP microscope (Marianas, 3i) with an excitation wavelength of 750 nm for NAD(P)H and 890 nm for FAD. Cells were cultured in MEGM media Kit (Lonza, Catalog No. CC-3150), prepared as described by the ATCC, and passaged at approximately 70% confluency. Following passaging, approximately 100 000 cells were seeded in 35 mm imaging dishes (Mattek). Cells were imaged after 1 h, 12 h, and 24 h following treatment.

Images were acquired with three frame summation, a dwell time of 50 *μ*s, and laser power below 5.0 mW at the sample, yielding at least 100 photons at the peak of the biexponential decay for each pixel. Raw FLIM data were fit using SPCImage 8.0 to a biexponential decay curve to extract NAD(P)H and FAD *τ*_m_, *τ*_1_, *τ*_2_, and *α*_1_ values following deconvolution with the system instrument response function (IRF), which was measured using a urea crystal second-harmonic generation signal at 900 nm. NAD(P)H intensity images were used to generate cell masks using the Cellpose 3.0 algorithm [22].

#### 3.1.2 Data Import

**Figure 7.**
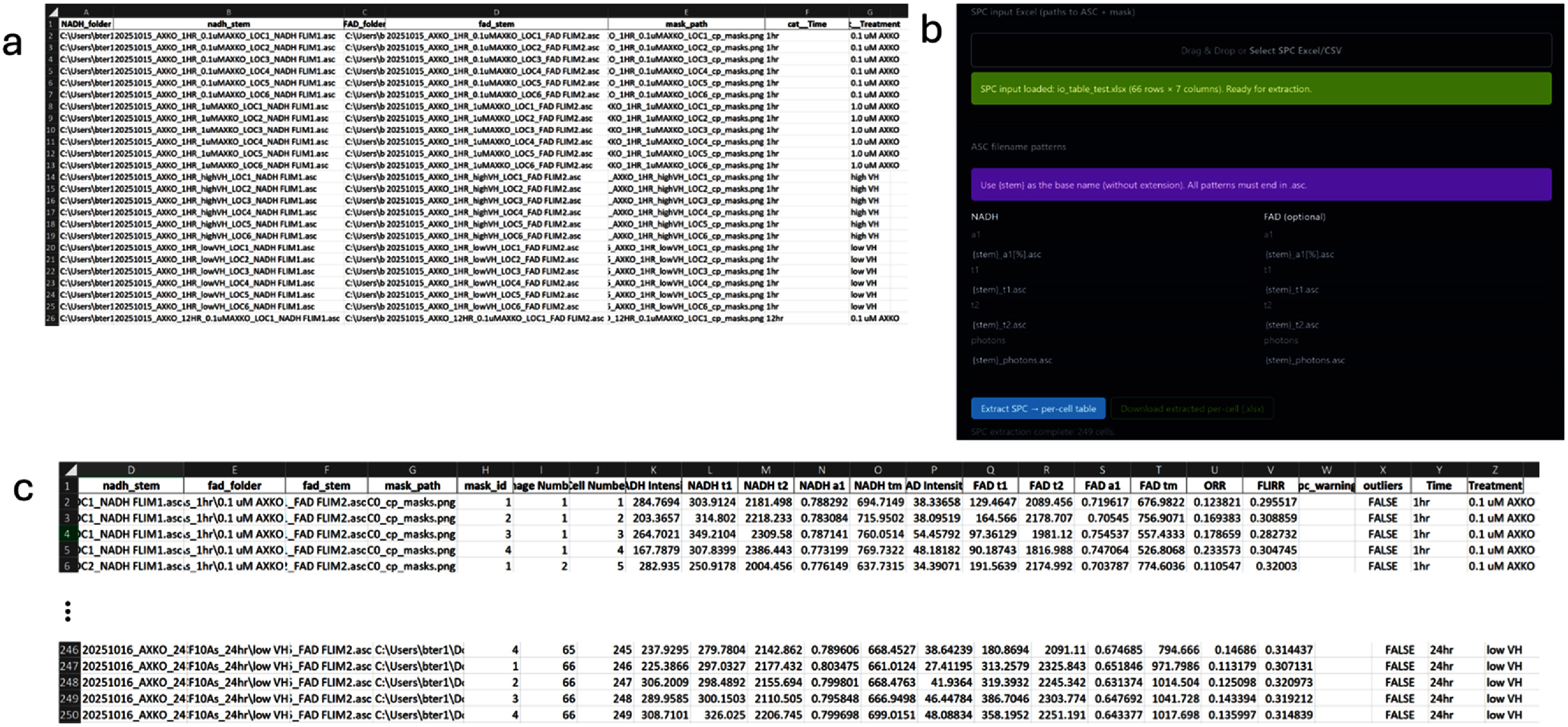
Import and per-cell FLIM endpoint extraction workflow in FLIMExplorer using MCF10A drug trial dataset. (a) Excel metadata file used for importing NAD(P)H, FAD, and cell mask image for per-cell FLIM endpoint extraction. (b) Import page for per-cell extraction and customization of FLIM ASCII image files. (c) Exported per-cell Excel output file generated from all imported FLIM image datasets using FLIMExplorer import page.

**Figure 8.**
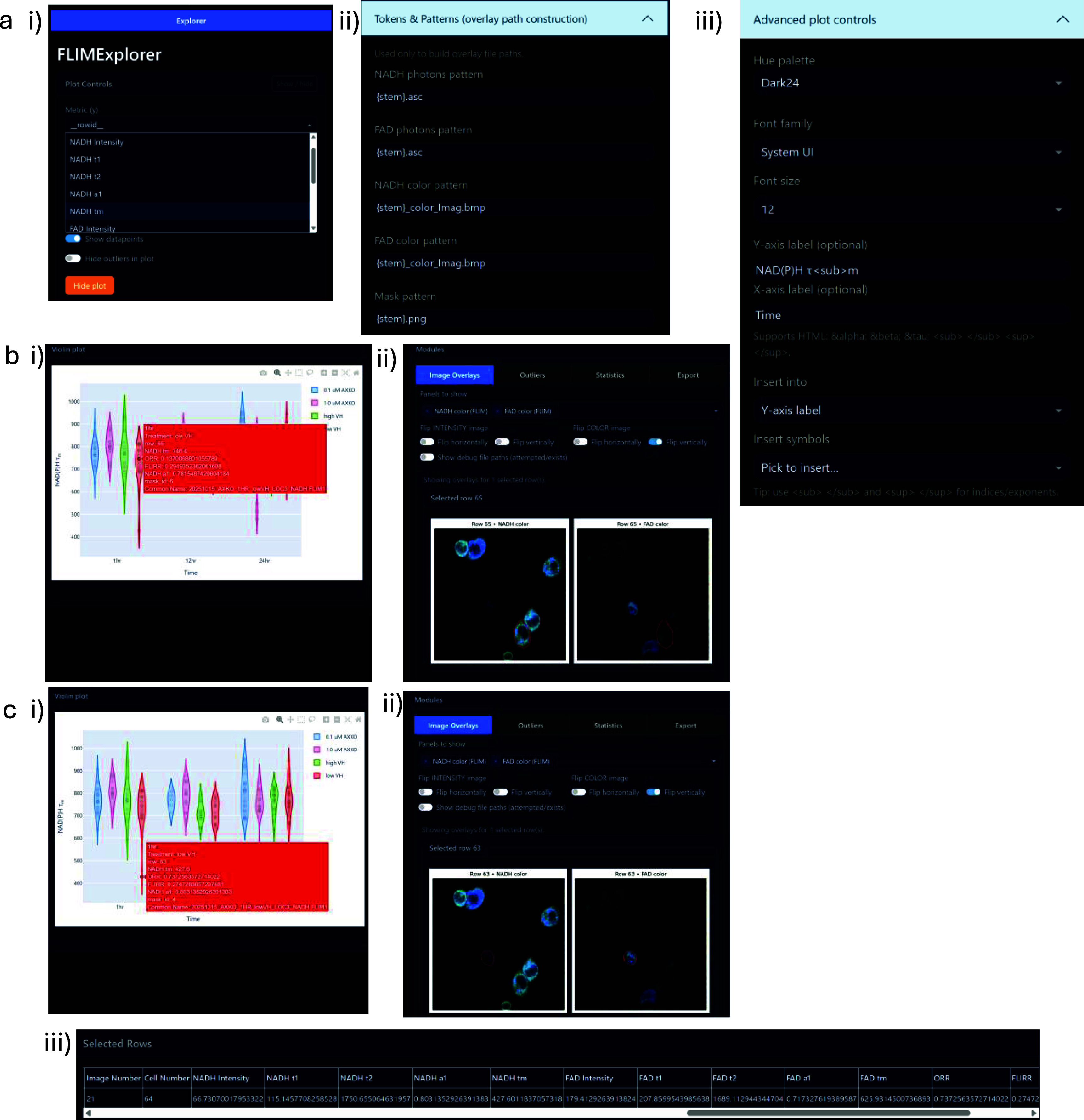
Customization of violin plots and data linkage with quantitative data and image objects of MCF10A dataset in FLIMExplorer. (a) Data visualization customization options within the explorer tab including: (i) Plot controls showing selectable FLIM metrics (*y*-axis) and independent variables primary and secondary variables from the input Excel file used as primary and secondary grouping variables (*x*-axis and hue), demonstrated here using time point and drug treatment group; (ii) customization of NAD(P)H, FAD, and cell mask input image filename patterns; and (iii) plot customization options including color palette, axis label font, font size, and formatted text (b)–(c) representative (i) interactive violin plot showing selected datapoints linked to (ii) corresponding NAD(P)H and FAD mean lifetime image object outlined in red. Hover information (i) displaying set of metadata and FLIM endpoint values displayed in red box.

Within the FLIMExplorer GUI, an Excel metadata file (figure 7(a)) specifying the NAD(P)H and FAD file paths and names, corresponding cell mask file locations, and the independent variables (time point and treatment group), was uploaded via the Import Tab. Cell-wise FLIM endpoints were extracted using the provided ASCII image files processed in SPCImage together with cell masks using customizable file naming options provided by the Import page (figure 7(b)). The resulting Pandas dataframe was then uploaded into FLIMExplorer page (figure 8) and could be exported as an Excel file for optional downstream analysis (figure 7(c)).

#### 3.1.3 Data visualization and linkage

In the Explorer tab, once FLIM endpoints were extracted and uploaded in the main stored data frame, NAD(P)H mean lifetime (*τ_m_*) was selected as the metric in ‘Plot Controls’ panel (figure 8(a)(i)). Time point and treatment group were specified as the primary and secondary grouping variables, respectively, generating a violin plot of the selected endpoint (figure 8(b)(i)). For linkage between quantitative data and the corresponding object image, NAD(P)H and FAD intensity and lifetime color images generated from SPCImage outputs could be customized using the ‘Tokens and Patterns’ settings (figure 8(a)(ii)). Plot appearance could be customized in the ‘Advanced Plot Controls’ panel, including font, font size, and color palette selection (figure 8(a)(iii)).

Hovering over an individual datapoint displayed a set of the corresponding FLIM endpoints values (figure 8(b)(i), red box). Selecting a datapoint simultaneously displayed the corresponding NAD(P)H and FAD intensity and mean lifetime color images, with the selected cell outlined in red (figure 8(b)(ii)). A potential non-viable cell exhibiting a low mean NAD(P)H lifetime (*τ*_m_) was identified directly from the violin plot (figure 8(c)(i)) and visualized in the ‘Image Overlay’ module (figure 8(c)(ii)), enabling quality control of individual datapoints directly from bulk quantitative datasets.

#### 3.1.4 FLIMExplorer endpoint and image linkage validation

**Figure 9.**
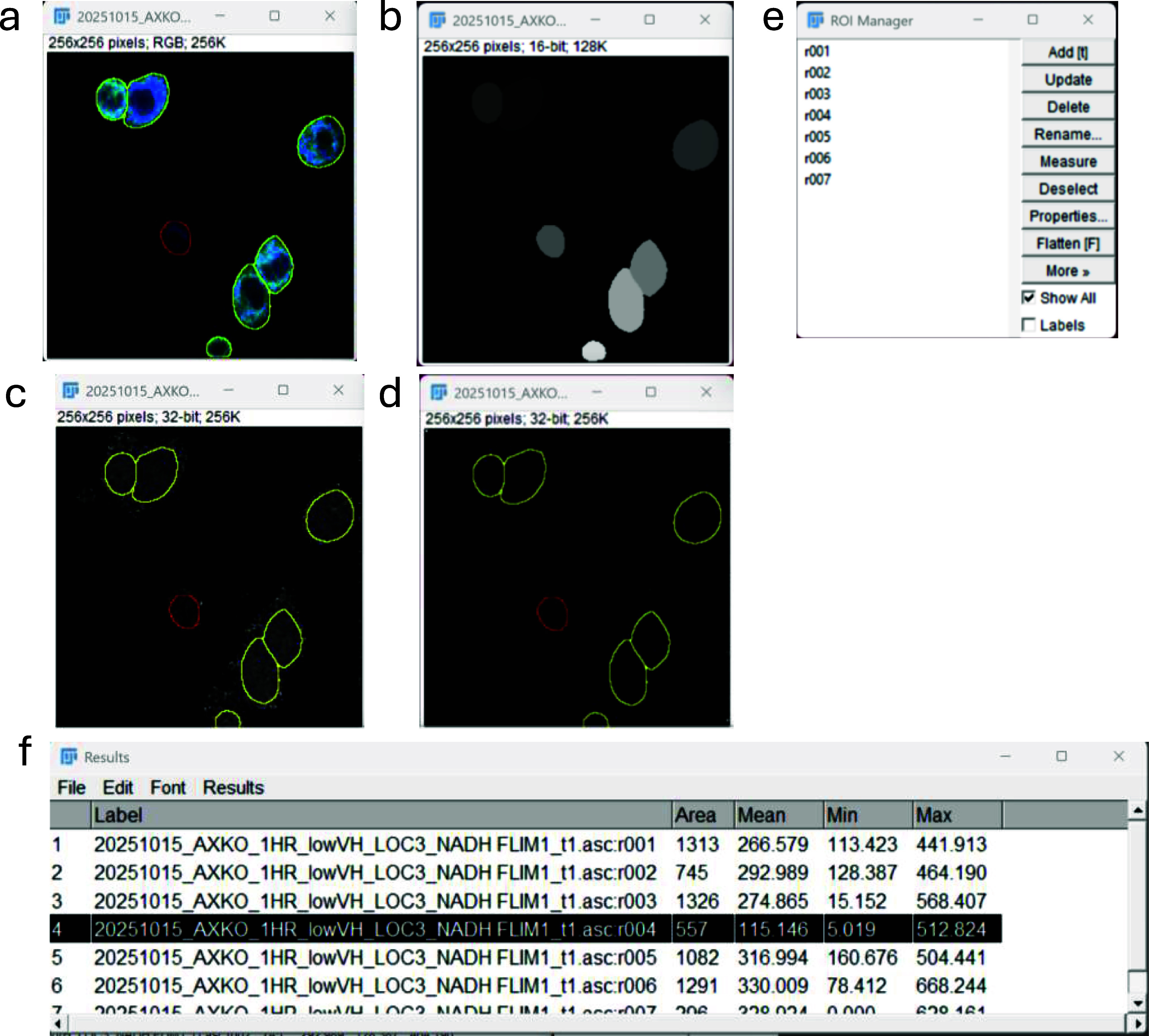
Comparison of workflow and verification of FLIMExplorer for linking image objects with ImageJ/FIJI. (a) Color NAD(P)H mean lifetime (τ_m_) image with outlined masks from ROI manager, (b) integer cell masks, (c) short lifetime (τ_1_) and (d) long lifetime (τ_2_) ASCII image files with cell mask outlines, (e) ROI manager generated from MorphLibJ plugin, and (f) results from measuring ROI objects using in ROI manager.

FLIMExplorer image linkage and per-cell endpoint extraction were validated against a conventional ImageJ/FIJI-based workflow. Using standard methods, linkage between a datapoint and its corresponding image object required manual identification of the image filename and cell integer mask value from the exported per-cell Excel dataset, requiring at least three distinct programs. The corresponding NAD(P)H *τ*_m_ image (figure 9(a)) and integer cell mask (figure 9(b)) are then opened in ImageJ/FIJI and the matching cell integer value was manually identified within the mask image. To validate quantitative agreement, the ROI manager in ImageJ/FIJI was used to view and measure the mean *τ*_1_ (figure 9(c)) and *τ*_2_ (figure 9(d)) values for the desired individual cell. This required converting the integer mask to ROIs using the MorphLibJ plugin [28] (figure 9(e)). The resulting values (figure 9(f)) matched those generated by FLIMExplorer (*τ*_1_, figure 8(c)(iii)), confirming accurate per-cell endpoint extraction and image-object linkage.

#### 3.1.5 Dataset 1: quality control and statistical testing

**Figure 10.**
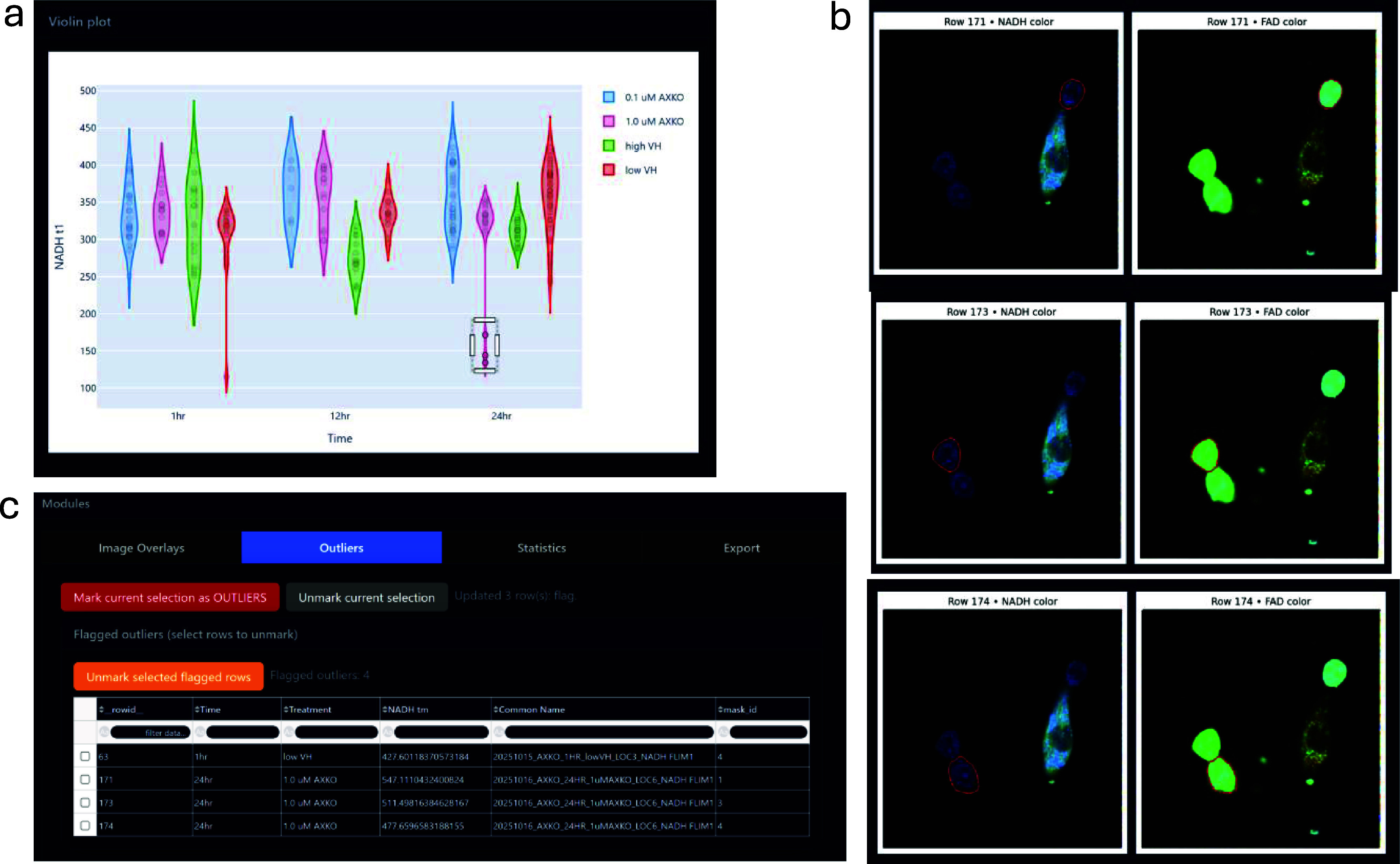
Outlier identification in FLIMExplorer. (a) Violin plot of NAD(P)H *τ*_1_ showing low-value outlier populations in the low-dose vehicle 1 h and 1.0uM AXKO 24 h groups. (b) Linked NAD(P)H and FAD lifetime images corresponding to selected low NAD(P)H *τ*_1_ datapoints. (c) Outlier module used to flag selected datapoints for downstream inclusion, exclusion for separate quantification.

FLIMExplorer enabled identification and flagging of potential non-viable cells as outliers for further downstream analysis. After selecting NAD(P)H *τ*_1_ as the displayed metric (figure 10(a)), low *τ*_1_ values observed in the low-dose vehicle at 1 h and in the 1.0 *μ*M AXKO treatment group at 24 h showed low NAD(P)H *τ*_1_ and elevated FAD *τ*_1,_ indicating potentially non-viable cells (figure 10(b)). Using the ‘Outliers’ module (figure 10(c)), selected datapoints could be flagged as outliers (figure 10(c)), allowing them to either be quantified separately or excluded from downstream statistical analysis. The full workflow is shown in video 1 (supplementary material).

**Figure 11.**
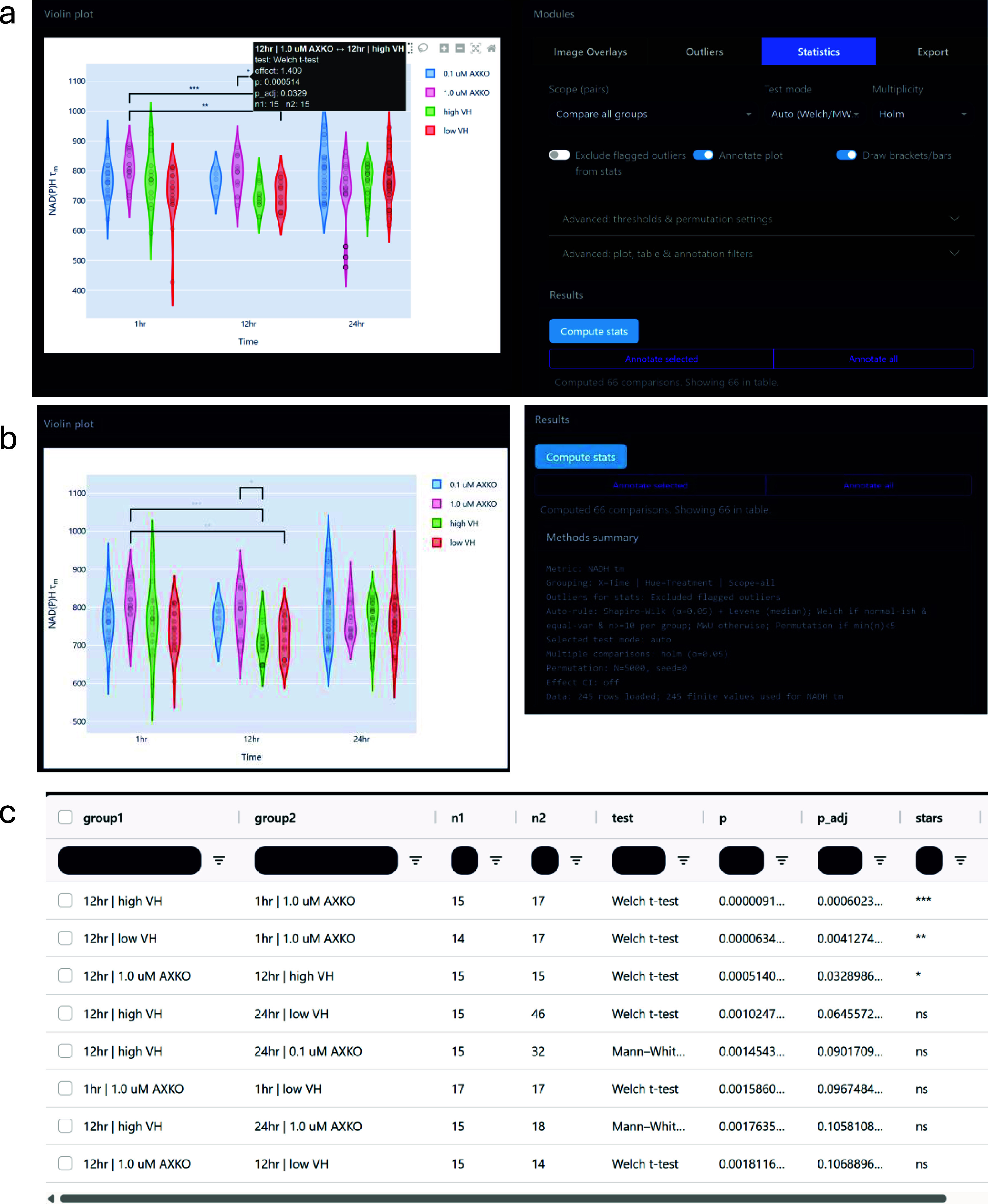
Statistical analysis workflows in FLIMExplorer with and without flagged outliers. (a) Violin plot annotated with statistical testing results. Gray box appears when hovering over statistical annotations after statistical testing is performed. (b) Violin plots without outlier data points and statistical annotations and (c) full statistical results table excluding flagged outliers.

FLIMExplorer additionally enabled customizable statistical analysis workflows including or excluding flagged outliers. Pairwise comparisons between all groups were automatically performed. Welch’s *t*-test or Mann-Whitney *U* tests were applied as appropriate, followed by Holm correction for multiple comparisons. Statistical analysis was performed both including outliers (figure 11(a)) and excluding flagged outliers (figure 11(b)). Resulting p-values were displayed in the statistical testing table (figure 11(c)) and annotated directly on the violin plots. Hovering over statistical significance annotations displayed the corresponding pairwise comparison results and adjusted p-values (figure 11(a), gray box). Together, these results demonstrate accurate linkage between per-cell FLIM endpoints and image objects, enabling rapid quality control and validation of outlier populations directly from bulk quantitative data.

### 3.2 Dataset 2: macrophages isolated from whole blood

The second dataset consisted primarily of peripheral blood mononuclear cells (PBMCs)-derived macrophage cultures isolated from whole blood, with residual non-macrophage cell populations also present. This dataset was acquired and organized by a different user, further demonstrating the generalizability of the software across users and data organization workflows. Macrophages were isolated from PBMCs separated from whole blood and imaged at Days 1, 3, 6, and 8 post-isolation. Cells were plated in 35 mm imaging dishes with RPMI 1640 cell culture media supplemented with 10% heat inactivated FBS and 2 ng ml^−1^ human macrophage colony-stimulating factor and incubated at 85% humidity, 5% CO_2_, and 37 °C until imaging. Images were acquired with the same parameters described for Dataset 1.

#### 3.2.1 Data import

**Figure 12.**
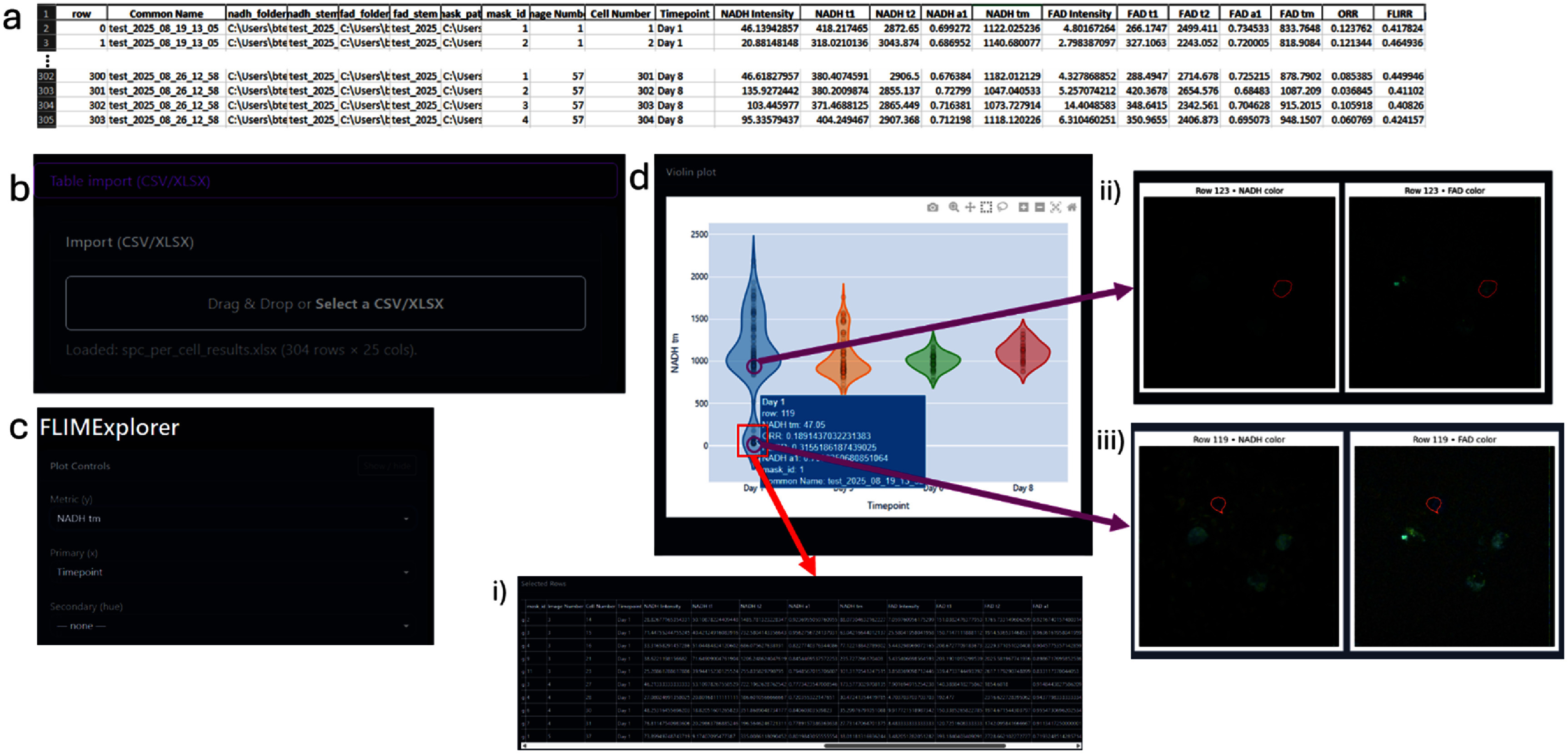
Data import using input Excel file with tabulated per-cell FLIM endpoints of macrophages from isolated PBMC from whole blood. (a) input Excel data (b) table import, (c) selection of metric and primary independent variable NAD(P)H mean lifetime *τ_m_* (d) violin plot of NAD(P)H *τ_m_* showing (i) all metrics loaded to GUI from multi-select tool outputs and images linkage indicating (ii) representative macrophage cell and (iii) possible outlier including red blood cell.

Due to differences in file organization systems between individuals, NAD(P)H and FAD FLIM filenames were not directly matched as in Dataset 1. Therefore, an Excel metadata file was generated with the NAD(P)H and FAD file names to be matched. A Python script, generated using OpenAI ChatGPT based on the user’s file organization structure, was used to expedite creation of the metadata file. Files were organized into separate folders for each time point, with additional NAD(P)H and FAD subfolders. Per-cell FLIM metrics had been previously extracted using MATLAB (figure 12(a)). Therefore, cell-wise FLIM metrics were imported directly using the ‘Table import’ module in the Import page (figure 12(b)).

#### 3.2.2 Data visualization and linkage

After selection of the desired FLIM metric, *α*_1_, within FLIMExplorer (figure 12(c)), heterogeneous cell populations present within the dataset could be visualized, particularly at Day 1 post-isolation, where platelets and RBCs remained present alongside macrophages (figure 12(d)). Although size filtering may be applied during cell mask generation, remaining outliers in the dataset could still arise from non-macrophage cell populations and therefore would need to be flagged. By Day 3, post-isolation, contaminating cell populations were substantially reduced for the remaining timepoints.

### 3.3 Dataset 3: isolated CD3+ T cells

#### 3.3.1 Cell preparation and imaging acqusition

To validate compatibility with data acquired using different imaging systems and file organization workflows, a previously acquired CD3+ T cell dataset was analyzed using FLIMExplorer [27]. Thirty-five images of control and activated CD3+ T cells isolated from whole blood from a single donor were acquired using an Ultima (Bruker Fluorescence Microscopy) with a 100X/1.45 NA objective (Nikon Plan Apo Lambda), as previously described in Walsh *et al* [27].

#### 3.3.2 Data import

**Figure 13.**
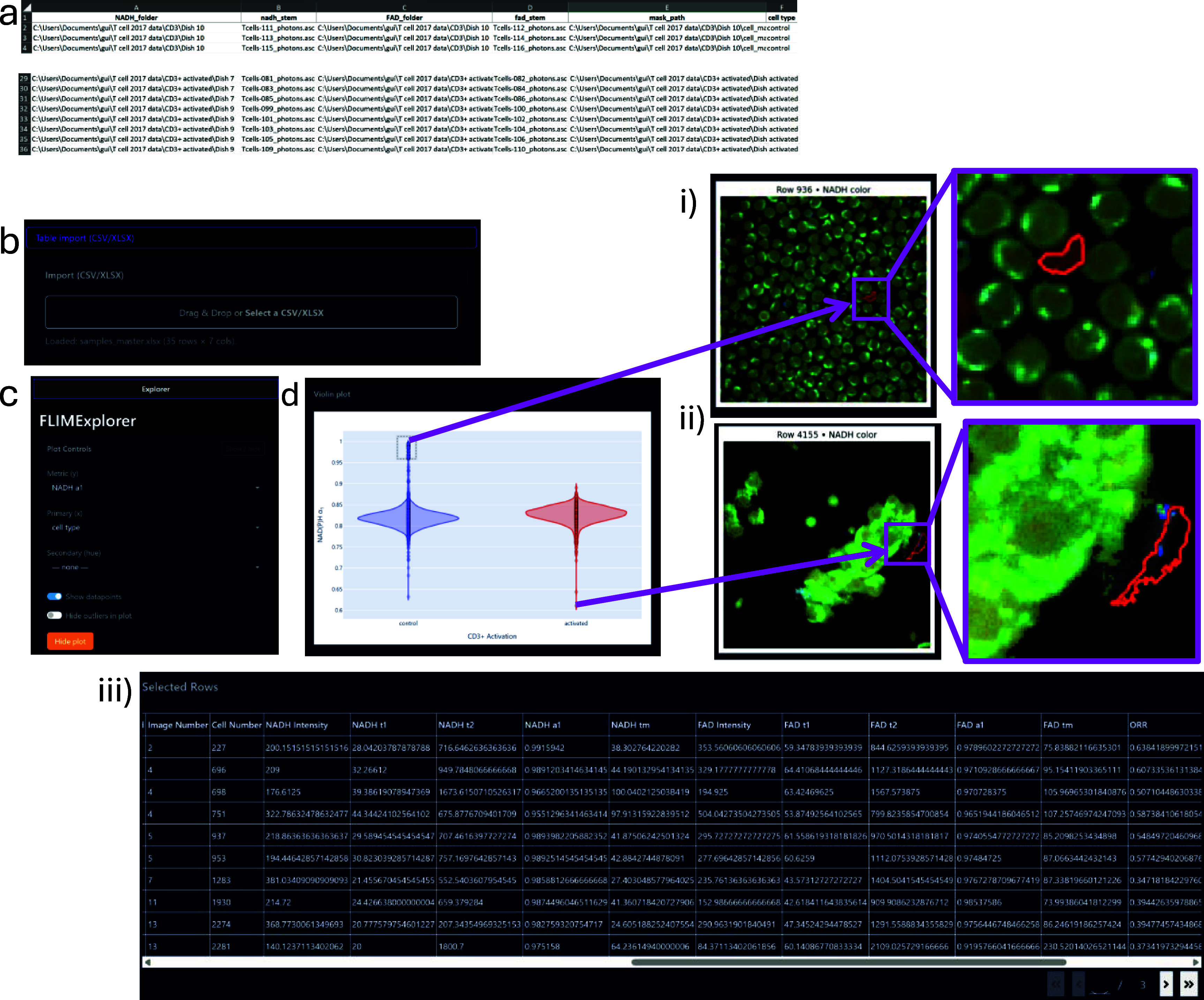
FLIMExplorer analysis of CD3+ T cell data acquired using a separate MP imaging system. (a) Excel manifest file containing paired SPCImage ASCII exports and corresponding cell mask filenames used for GUI import. (b) Successful import and extraction of SPCImage ASCII files together with CellProfiler-generated TIFF cell masks within FLIMExplorer. (c) Plot controls panel showing selection of NAD(P)H *α*_1_ for visualization of activated and control CD3+ T cell data. (d) Interactive violin plots demonstrating linkage between quantitative FLIM data and corresponding image objects, including: (i) identification of an outlier cell within the control group, (ii) linkage of activated CD3+ T cell datapoints to corresponding image objects, and (iii) multi-selection of datapoints with elevated α_1_ values within the control group (gray box).

NAD(P)H and FAD image filenames were not directly matched; therefore, an Excel manifest file was created with folder stems and file names of paired NAD(P)H, FAD, and corresponding masks with a Python script generated with OpenAI ChatGPT to automate file pairing. A total of 4523 cells were included in the dataset. The manifest Excel file was uploaded to the GUI import page, and the cell-wise values were extracted from SPCImage ASCII exports (figure 13(a)).

#### 3.3.3 Data visualization and linkage

In the FLIMExplorer page, NAD(P)H *α*_1_ data was selected for visualization, and the corresponding lifetime color images were successfully linked to the individual datapoints within the violin plots (figure 13(b)). FLIMExplorer allowed visualization of outlier cell objects and small segmented fragments that were not removed during prior preprocessing steps previously (figure 13(c)). These datapoints could be flagged as outliers and optionally excluded from downstream analysis. Statistical testing was performed twice, including and excluding flagged outliers (figure 13(d)). These findings demonstrate compatibility of FLIMExplorer across multiple imaging systems, segmentation workflows, and experimental users.

### 3.4 Code metadata

**License Type:** MIT License

**Programming Language:** Python

**Software versioning info:** Python 3.10 or higher

**Dependencies and installation instructions:** This software can be installed using the provided YAML environment file in the GitHub repository, specifying the Python version (3.10) and all libraries and specific versions. To install the software, the user creates the Conda environment using the configuration file and activates the dedicated environment. The program can then be run using ‘python flim-explorer-main.py’ in the command line. Upon execution, a Dash local server will be initialized, accessible via the provided URL to open the interactive GUI in a standard web browser.

**Validation and testing protocols:** The software was tested on three datasets (MCF10A cells, heterogenous mixture of macrophages and blood cells isolated from whole blood samples, and isolated T cells) acquired with the two independent MP FLIM microscope. FLIM endpoint averaging per cell was validated using previously published MATLAB code [26]. Sample data sets are provided in the GitHub repository to allow for testing of the software.

**Reproducibility guidelines:** The software does not modify input data files and allows the user to export all derived results, including averaged FLIM endpoints and statistical tests for record-keeping.

A fixed Conda environment ensures reproducible data analysis across different systems by ensuring identical library versions.

## 4 Conclusion

With the increasing need for single-cell analysis techniques in the field of quantitative image analysis, the FLIM community lacks widely-adopted tools to standardize FLIM processing and analysis. We have developed FLIMExplorer, an interactive GUI application for per-object visualization and analysis of FLIM data. This application enables users to link quantitative data to cell images for data exploration and outlier analysis. Using a web-based Dash application, users can upload either FLIM metadata files or object-averaged FLIM endpoints along with the corresponding intensity and FLIM color images for visualization and statistical testing. Data selection allows for linking data points to the corresponding exact cell. Outliers can be identified and flagged, allowing for more transparent data filtering. Moving forward, this software can be improved by making it more generalizable by allowing other input FLIM file types from FLIM acquisition software. Together, these features position FLIMExplorer as an accessible downstream application tool for single-cell FLIM analysis and the exploration of cellular heterogeneity that can be incorporated into existing FLIM workflows, facilitating standardized data processing for the FLIM community.

## Data Availability

The data that support the findings of this study are openly available at the following URL/DOI: https://github.com/walshlab/FLIM_Explorer/tree/main/sample_data [26]. FLIMExplorer Workflow available at https://doi.org/10.1088/2515-7647/ae7da3/data1.
